# A Large Retrospective Observational Study of Nonpharmacologic Treatment Use Among German Patients Receiving Long‐Term Opioid Therapy for Chronic Noncancer Pain

**DOI:** 10.1155/prm/2607538

**Published:** 2026-04-29

**Authors:** Nils Frederik Schrader, Anja Niemann, Milena Weitzel, Carina Abels, Nikola Blase, Anja Neumann, Cordula Riederer, Joachim Nadstawek, Wolfgang Straßmeir, Jürgen Wasem, Silke Neusser

**Affiliations:** ^1^ Institute for Healthcare Management and Research, University of Duisburg-Essen, Essen, Germany, uni-due.de; ^2^ DAK-Gesundheit, Hamburg, Germany; ^3^ Association of German Doctors and Psychotherapists Practicing in Pain Medicine and Palliative Care (BVSD e.V.), Berlin, Germany

## Abstract

**Introduction:**

Evidence‐based practice guidelines recommend combining the long‐term use of opioids for chronic noncancer pain (CNCP) with suitable nonpharmacologic treatments. Whether long‐term opioid therapy (LTOT) in Germany aligns with these recommendations is unknown so far. Therefore, our objective was to determine the use of nonpharmacologic treatments among patients receiving LTOT for CNCP.

**Methods:**

The retrospective observational analysis was based on administrative claims data of the German statutory health insurance fund *DAK-Gesundheit*. Patients with LTOT and no cancer diagnosis were selected between January 2018 and June 2019. The utilization of nonpharmacologic treatments, including physical therapy, psychotherapy, lifestyle modification, and other inpatient and outpatient treatments, was examined over a two‐year period. Subgroup analyses were conducted by age, preindex opioid prescriptions (≥ 1 year vs < 1 year), and the presence of ICD‐10 diagnoses, including osteoarthritis, chronic back pain, inflammatory rheumatic diseases (excluding rheumatoid arthritis), neuropathy/polyneuropathy, radiculopathy, and chronic pain disease. Additionally, regression analyses were performed to examine the association between diagnostic groups and the utilization of treatments.

**Results:**

A total of 113,476 patients met the inclusion criteria, of whom 25,945 (23%) did not utilize any of the defined nonpharmacologic treatments during the two‐year observation period. The most frequently utilized treatment was physical therapy (62%). Psychotherapy was used by 7% of patients, outpatient special pain therapy by 16%, whereas lifestyle modification (relaxation exercises and nutrition therapy) was used by fewer than 30 patients. Patients with ≥ 1 year of opioid prescriptions prior to inclusion and elderly patients utilized most treatments less frequently. Over the 2‐year period, treatment provision remained largely constant, except for a steady decline in physical therapy for patients who had initiated opioid therapy more recently. Except for chronic pain disease, certain diagnoses were only weakly associated with treatment utilization.

**Conclusions:**

Contrary to guideline recommendations, a notable proportion of LTOT patients may not receive pain management combining opioid therapy with nonpharmacologic treatments, particularly elderly patients and those with longer treatment duration. Future research should examine reasons for the low use of nonpharmacologic treatments.

**Trial registration:** German Clinical Trials Register: DRKS00024854

## 1. Introduction

Chronic noncancer pain (CNCP) is a common health concern. In Germany, the prevalence of CNCP is estimated to range between 17% [1] and 24% [2]. Several studies have observed a rise in the number of patients receiving opioid analgesics for CNCP in Germany over the past 2 decades [3–5]. However, scientific reviews suggest that there is uncertainty as to whether long‐term opioid therapy (LTOT) of CNCP provides relevant pain relief [6–9], although it may be appropriate in selected cases [10]. LTOT has been associated with a range of adverse events, including myocardial infarction, abuse, and overdose [6].

CNCP can be effectively treated with multimodal and multidisciplinary interventions grounded in the biopsychosocial model of pain [11–13]. The use of such interventions is further recommended in clinical guidelines for chronic pain and opioid therapy [14–18]. In particular, the German guideline on LTOT for CNCP recommends that opioid therapy should be complemented by nonpharmacologic treatments. The selection of appropriate nonpharmacologic treatments should be based on the guidelines for the underlying medical indication and may include self‐help services, physical therapy, lifestyle modification, or psychotherapy [15]. A multitude of such nonpharmacologic, noninvasive therapeutic modalities have been shown to result in at least temporary improvements in pain and/or function for selected chronic pain conditions, while simultaneously exhibiting a low risk of adverse events. These include exercise therapy, spinal manipulation, massage, acupuncture [19, 20], and psychotherapeutic interventions, such as cognitive behavioral therapy [19–21], dietary changes [22], and mindfulness‐based stress reduction [19, 20].

To our knowledge, no German study has previously investigated the extent to which LTOT patients receive nonpharmacologic interventions and which patient groups receive only opioid therapy. Therefore, the objective of this study was to address this knowledge gap and (i) determine the utilization of nonpharmacologic treatments among patients receiving LTOT for CNCP using German health insurance data. Furthermore, the study aimed to (ii) examine the treatment utilization over the course of therapy and (iii) within patient groups based on age, duration of therapy, and pain diagnoses. This study was part of the Op‐US project, which was publicly funded by the Innovation Fund of the German Federal Joint Committee (G‐BA; funding No. 01VSF19059).

## 2. Methods

### 2.1. Data Selection

The data set was derived from the administrative claims data of the statutory health insurance (SHI) fund *DAK-Gesundheit* and spanned the period from January 2018 to March 2021. In 2018, the *DAK-Gesundheit* insured approximately 5.7M individuals nationwide [23]. A detailed description of the inclusion criteria and the study design has been previously published [24], and other results based on the same dataset have been reported elsewhere [25]. A depiction of the selection process and the observation period can be found in Supporting Figure 1.

In summary, the inclusion criteria were at least one outpatient prescription of an opioid analgesic in each of two consecutive quarters between January 2018 and June 2019 (selection period); no signs of cancer or palliative care during the selection period; and age ≥ 18 years at the time of inclusion. The first prescription during the selection period is hereinafter referred to as the index prescription. The following codes from the Anatomical Therapeutic Chemical (ATC) classification system were used to identify opioid analgesics in outpatient prescription data: N02AA01 (morphine), N02AA03 (hydromorphone), N02AA05 (oxycodone), N02AA55 (oxycodone/naloxone), N02AB03 (fentanyl), N02AE01 (buprenorphine), N02AX51 (tilidine/naloxone), N02AX02 (tramadol), N02AJ13 (tramadol/paracetamol), and N02AX06 (tapentadol). We required one prescription in each of two consecutive quarters, and a minimum 90‐day interval between the index prescription and the last prescription during the observation period to align with the definition of LTOT (≥ 3 months) [15]. For each patient, the observation period began at the date of the index prescription and ended 2 years later or at death. To allow a complete two‐year follow‐up within the available data, the latest eligible index prescription had to be issued in March 2019, followed by a prescription in the subsequent quarter.

In addition to drug prescriptions, the claims data captured information on SHI‐covered healthcare utilization, including procedure and diagnosis codes from outpatient physician visits and inpatient stays, as well as prescriptions for therapeutic services (e.g., physical therapy, occupational therapy) and assistive devices (e.g., wheelchairs, hearing aids).

### 2.2. Variable Specification and Research Design

#### 2.2.1. Selection of Treatments

In order to ascertain which specific nonpharmacologic treatments from SHI standard care should be included, a search was conducted of the directories for item numbers of the National Association of SHI funds [26–28], the SHI doctor fee schedule [29], and medical procedure classification [30] for services related to pain management. Included services are shown in Table 1.

**TABLE 1:** List of included nonpharmacologic treatments and services.

Treatment recommended in LTOT guideline	Included treatments	Identifier
Physical therapy	Therapeutic exercise	PNr. x05xx‐x08xx
	Manual therapy	PNr. x12xx
	Electrotherapy	PNr. x13xx, 865xx
	Lymphatic drainage	PNr. x02xx, 861xx
	Massage	PNr. x01xx, 860xx
	Thermotherapy	PNr. x15xx, 866xx
	Other physical therapy:	
	• Movement therapy	PNr. x03xx, x04xx, 862xx, 863xx
	• Movement bathing	PNr. x09xx, x10xx
	• Medical bathing	PNr. x17xx, 868xx, 869xx
	• Hydrotherapy	PNr. x16xx, 867xx
	• Light therapy	PNr. x14xx
	• Extension/traction	PNr. x11xx, 864xx

Lifestyle modification	Relaxation exercises	PNr. 8401, 8406
	Nutrition therapy	PNr. x50xx

Psychotherapy	Psychotherapeutic consultation hour	GOP 35151
	Psychotherapeutic treatment:	
	• Trial sessions (max. 4)	GOP 35150
	• Depth psychology founded psychotherapy	GOP 35401, 35402, 35405, 35503–35509, 35513–35519
	• Analytical psychotherapy	GOP 35411, 35412, 35415, 35523–35529, 35533–35539
	• Behavioral therapy	GOP 35421, 35422, 35425, 35543–35549, 35553–35559
	• Acute treatment sessions	GOP 35152
	Diagnostic measures	GOP 3560×
	Other psychotherapy:	
	• Psychotherapeutic interview	GOP 23220
	• Relaxation techniques	GOP 35111–35113
	• Hypnosis	GOP 35120
	• Report on possible initiation of short‐term therapy	GOP 35130
	• Report on possible initiation of long‐term therapy	GOP 35131
	• Biographical anamnesis	GOP 35140
	• In‐depth diagnostics	GOP 35141
	• Surcharge for neurological or psychiatric findings	GOP 35142

*Other services*		
Outpatient care	Psychosomatic care	GOP 35100, 35110
	Special pain therapy	GOP 30700, 30702, 30704
	Acupuncture	GOP 30790, 30791

Hospital care	IMPT	OPS 8–918, 8‐91b, 8–91c
	Other pain therapy	OPS 8–910 to 8–917, 8–919

Nonpharmaceutical prescriptions	Prescription of TENS device	PNr. 09.37.01.xxxx

*Note:* GOP doctor fee schedule; PNr. item number; OPS procedure classification.

Abbreviations: IMPT = interdisciplinary multimodal pain therapy, TENS = transcutaneous electrical nerve stimulation.

In a first step, the directories were searched for nonpharmacologic therapeutic procedures explicitly recommended for outpatient care by the German LTOT guideline. These included physical therapy, lifestyle modification, psychotherapy, and self‐help services [15]. The identified physical therapies encompassed several services, such as therapeutic exercise, manual therapy, and massage. The included psychotherapy services were psychotherapeutic consultation hours, diagnostic and treatment measures, such as depth psychology, analytical psychotherapy, and behavioral therapy. Relaxation exercises and nutrition therapy were classified as components of lifestyle modification. Self‐help services were not included in the data because they do not fall within the scope of the standard SHI services.

Furthermore, based on the directory search, other services with pain therapy components not listed in the LTOT guideline were identified. These included outpatient services, such as psychosomatic care, special pain therapy, acupuncture, hospital services such as interdisciplinary multimodal pain therapy (IMPT), and the prescription of transcutaneous electrical nerve stimulation (TENS) devices (see Table 1). Psychosomatic care represents a further training for general practitioners and specialists, with the objective of enhancing diagnostic and communication skills for psychosomatic conditions and fostering collaboration in psychotherapeutic care [31]. Services were subsumed under special pain therapy if they were provided as part of a quality assurance agreement with the regional association of SHI physicians. The agreement provides for the acquisition of pain‐centered qualifications and therapeutic equipment; special pain therapy may include physical and psychotherapeutic or multimodal methods [32]. IMPT is a treatment program for CNCP that follows the biopsychosocial model of pain management, combining approaches from different disciplines, such as behavioral and physical therapy [33]. In German SHI standard care, IMPT is typically carried out in a hospital setting and requires the involvement of multiple medical disciplines, including at least one psychiatrist, psychotherapist, or psychosomatic specialist, under the supervision of a physician with additional qualification in pain therapy. The procedure can be delivered as short‐term inpatient (max. 6 days), inpatient (≥ 7 days), or day patient (individual days) [34]. Moreover, IMPT is recommended in the German guideline on low back pain, e.g., for patients with continuous pain progression or significant psychosocial comorbidities [35].

For each service, we determined the proportion of patients with at least one utilization during the 2‐year observation period, as well as the proportion of patients who did not utilize any of the listed services throughout this period.

#### 2.2.2. Utilization Over the Course of Observation Period

To assess the longitudinal utilization of nonpharmacologic treatments during opioid therapy, the two‐year observation period of each patient was divided into eight consecutive 91‐day intervals (follow‐up quarters). Because not all patients necessarily received opioid prescriptions in every interval, the number of patients who continued opioid therapy was determined separately for each follow‐up quarter. Patients were excluded from the subsequent quarters if they either (i) died during the preceding quarter or (ii) received their final opioid prescription at least 90 days prior to the subsequent quarter. For each follow‐up quarter, the proportion of patients with at least one utilization was calculated among those who continued opioid therapy in that quarter.

#### 2.2.3. Utilization According to Medical Diagnosis

The German guideline on LTOT for CNCP contains an evidence‐ and consensus‐based list of various pain conditions and corresponding ICD‐10 diagnoses potentially eligible for long‐term use of opioid analgesics [15, 36]. The tracer ICD‐10 codes were used to identify pain‐related outpatient and inpatient diagnoses in the claims data. In case the guideline did not specify any ICD‐10 codes for a pain condition, the codes were identified through a literature review and then discussed among the coauthors with clinical expertise. This resulted in a total of 18 guideline‐based diagnostic groups, which are listed in Supporting Table 1. Diagnoses were required to be present in the first two quarters of opioid prescription, with outpatient diagnoses only considered if they were secured.

The percentage of patients with at least one utilization of any treatment from Table 1 was determined in the five most frequent diagnostic groups found in the data: osteoarthritis pain, chronic back pain, chronic pain associated with inflammatory rheumatic diseases other than rheumatoid arthritis, any neuropathy or polyneuropathy, and painful radiculopathy (for ICD‐10 codes, see Supporting Table 1). In addition to the five most frequent guideline‐based diagnoses, a variable for the diagnosis of chronic pain disease (persistent somatoform pain disorder: ICD‐10 F45.41; other chronic pain: R52.2) was defined. As it is not possible to definitively attribute prescriptions to a specific diagnosis based on SHI claims data, patients may be assigned to more than one diagnostic group. Patients without any of the 18 diagnoses from the guideline or without chronic pain disease were classified as patients with no guideline‐based diagnosis.

#### 2.2.4. Patient Subgroups

The study population was grouped by the duration of opioid prescriptions recorded before the index prescription (preindex; see Supporting Figure 1) to distinguish newer patients from established patients. Patients included in the first quarter of 2018 with outpatient opioid prescriptions in all four calendar quarters of 2017 were classified as having ≥ 1 year of preindex opioid prescriptions; the remaining patients, including those selected after the first quarter of 2018, were classified as having < 1 year. Furthermore, patients were grouped into age groups 18–49 years, 50–69 years, 70–89 years, and ≥–90 years.

### 2.3. Statistical Analysis

Utilization was evaluated for the entire observation period and at temporal intervals using frequencies and percentages. In addition, five binary logistic regression models were specified to determine whether the utilization of nonpharmacologic treatments was related to specific pain diagnoses. Dependent variables in these models were utilization of physical therapy, psychotherapy, psychosomatic care, special pain therapy, and IMPT. The explanatory variables included the five most frequent guideline‐based diagnoses, diagnosis of chronic pain disease, and a variable indicating the absence of any guideline‐based diagnosis or chronic pain disease. Age, gender, preindex opioid prescriptions, and a dummy indicating discontinued opioid therapy were included as control variables.

Comorbidity may act as a confounding factor in healthcare utilization. Therefore, the regression model additionally included a score of up to 30 comorbidities based on the ICD‐10 codes from the Elixhauser Comorbidity Index as proposed by Quan et al. [37].

The models were evaluated for multicollinearity through the use of correlation matrices and variance inflation factors (VIFs). All VIFs were found to be below 1.8, indicating the absence of multicollinearity [38]. *p*‐Values were considered statistically significant below 0.05. All data preparation and analysis steps were conducted using Stata Version 17.0.

### 2.4. Ethical Approval and Trial Registration

The implementation of this study was approved by the ethics committee of the medical faculty of the University of Duisburg‐Essen in September 2021 (ref. no. 21‐9964‐BO) and registered in the German Clinical Trials Register in April 2021 (drks.de/search/en, identifier: DRKS00024854).

### 2.5. Use of AI Technology

We used DeepL Write and ChatGPT 5 for grammar and style editing and DeepL Translator for short translations into English. All scientific content, analyses, and interpretations were authored and verified by the authors; AI tools were used solely for linguistic assistance and were not used to generate, analyze, or alter research data or results.

## 3. Results

### 3.1. Study Population

The inclusion criteria were met by 113,476 adults who had been receiving opioid therapy for more than three months, representing approximately 2% of the insured population of *DAK-Gesundheit* in 2018 [23]. The cohort was approximately evenly split between patients with ≥ 1 year of preindex opioid prescriptions (49%) and those with < 1 year (51%). The average age was 71.8 years (± 14.4), with the majority being female (75%).

Within the first two quarters of observation, 41% of patients presented with ICD‐10 diagnoses of chronic osteoarthritis pain, 37% with chronic back pain, 34% with chronic pain associated with inflammatory rheumatic diseases other than rheumatoid arthritis, 18% with any polyneuropathy or neuropathy, and 17% with painful radiculopathy. Approximately 41% of the study population was diagnosed with chronic pain disease (ICD‐10 R52.2 or F45.41). More than 20% of the study population had no guideline‐based diagnosis or no diagnosis of chronic pain disease within the first two quarters.

### 3.2. Utilization of Nonpharmacologic Treatments

During the 2‐year observation period, almost one quarter of the included LTOT patients (23%) received none of the defined nonpharmacologic treatments and 64% received any treatment recommended in the LTOT guideline. Furthermore, 13% received no treatments recommended in the LTOT guideline but utilized other services pertinent to pain management (see Table 2).

**TABLE 2:** Overview of patient demographics and treatment utilization among *n* = 113.476 long‐term opioid patients.

	***n***	**%**

Female	84,605	74.6%

*Age groups*		
18–49 years	8571	7.5%
50–69 years	35,735	31.5%
70–89 years	60,136	53.0%
≥ 90 years	9034	8.0%

*Preindex opioid prescriptions*		
< 1 year	57,793	50.9%
≥ 1 year	55,683	49.1%

*Utilization of nonpharmacologic treatments within the observation period*		
No utilization	25,945	22.9%
Treatments recommended in the LTOT guideline^∗^	72,686	64.0%
Other nonpharmacologic treatments only^∗∗^	14,845	13.1%

Abbreviations: IMPT = interdisciplinary multimodal pain therapy, TENS = transcutaneous electrical nerve stimulation.

^∗^Utilization of physical therapy, psychotherapy, and lifestyle modification at least once.

^∗∗^Utilization of psychosomatic care, outpatient special pain therapy, IMPT, other pain therapy, or prescription of a TENS device, at least once, but no utilization of treatments recommended in the LTOT guideline.

Table 3 shows the frequency of treatment utilization at least once during the observation period. By far, the most frequently utilized treatment was physical therapy, which was received by 62% of the patients. This was predominantly in the form of therapeutic exercise (54%), followed by manual therapy (14%), thermotherapy (12%), and lymphatic drainage (12%). Lifestyle modification in terms of relaxation exercises or nutrition therapy was received by less than 30 patients (< 0.1%).

**TABLE 3:** Utilization of nonpharmacologic therapies at least once during the two‐year observation period.

Guideline‐recommended treatments	*n*	%
*Physical therapy*	70,531	62.2
Physiotherapy/medical gymnastics	61,449	54.2
Manual therapy	16,012	14.1
Thermotherapy	14,100	12.4
Lymphatic drainage	13,229	11.7
Massage	5805	5.1
Other physical therapy	1881	1.7
Electrotherapy	1732	1.5

Lifestyle modification^†^	< 30	< 0.1

*Psychotherapy*	8133	7.2
Psychotherapeutic treatment	4137	3.7
Psychotherapeutic consultation	4071	3.6
Other psychotherapy	3432	3.0
Diagnostic measures	3140	2.8

*Other services*		
Psychosomatic care (outpatient)	41,578	36.6
Special pain therapy (outpatient)	17,968	15.8
Acupuncture (outpatient)	7479	6.6
IMPT	3468	3.1
Other pain therapy (hospital)	8159	7.2
TENS prescription	3654	3.2

Abbreviations: IMPT = interdisciplinary multimodal pain therapy, TENS =  transcutaneous electrical nerve stimulation.

^†^Small numbers suppressed (*n* < 30) to protect privacy.

More than 7% of the patients received psychotherapy. The level of utilization was distributed almost equally across psychotherapeutic services, including 4% for psychotherapeutic consultation hours and psychotherapeutic treatments, respectively, as well as 3% for diagnostic measures and other psychotherapy. Outpatient psychosomatic care was utilized by 37%. Sixteen percent had special pain therapy, 7% had outpatient acupuncture sessions, and 3% received TENS prescriptions. Regarding hospital care, 3% received IMPT and 7% received other pain treatments.

### 3.3. Service Utilization Over the Course of the Observation Period

#### 3.3.1. Number of Continued Opioid Therapies

Figure 1 shows two plots: the number of patients who continued opioid therapy over the observation period, stratified by the duration of preindex opioid prescribing (Figure 1(a)) and age groups (Figure 1(b)). As shown in Figure 1(a), a slightly greater number of patients with < 1 year of preindex opioid prescriptions were receiving opioids in the first follow‐up quarter (Q1) than those with ≥ 1 year (57,792 vs. 55,684). However, by the final follow‐up quarter (Q8), a greater number of patients with ≥ 1 year of preindex prescriptions remained on therapy (36,750 (64%) vs. 47,547 (85%)). As shown in Figure 1(b), among the 9034 patients aged 90 years and older, 5432 (60%) continued to receive opioid therapy until the final follow‐up quarter. This proportion was notably higher in the groups of patients aged 70–89 years (44,255/60,136; 74%), 18–49 years (6433/8571; 75%), and 50–69 years (28,177/35,735; 79%).

FIGURE 1Number of patients in opioid therapy, stratified by the duration of preindex opioid prescribing (a) and age groups (b).

**Figure:**
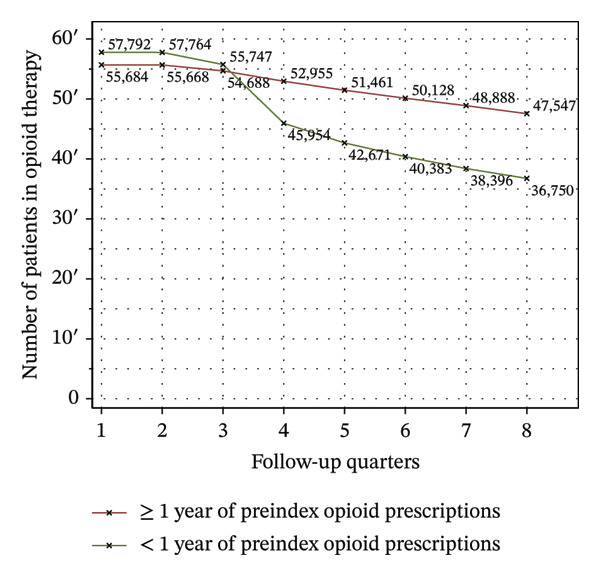
(a)

**Figure:**
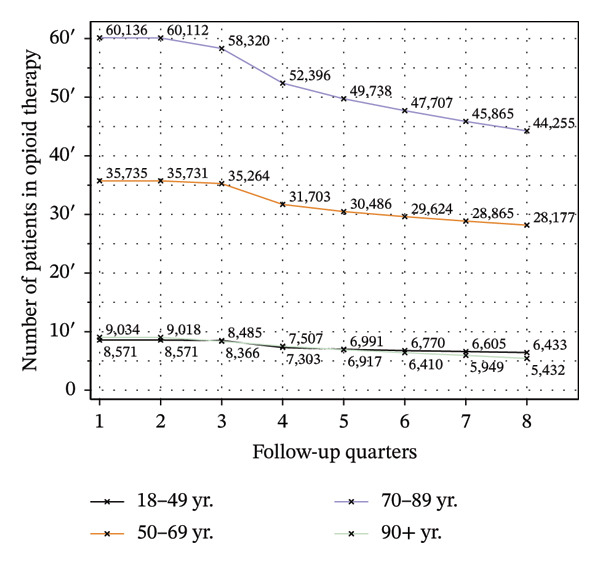
(b)

#### 3.3.2. Utilization Over the Course of Therapy

Figure 2 shows the quarterly utilization of nonpharmacologic treatments among patients who continued opioid therapy in the respective quarter, stratified by the duration of preindex opioid prescribing. In the initial follow‐up quarter, the proportion of patients without utilization was higher among those with ≥ 1 year of preindex opioid prescriptions (53% vs 44%, see Figure 2(a)). Over the course of the observation period, the proportion remained relatively stable within this group. However, for patients with < 1 year of preindex opioid prescriptions, the proportion increased until the fifth quarter, after which it remained at a comparable level as that of patients with ≥ 1 year (53% vs. 54% in Q8).

FIGURE 2Utilization of nonpharmacologic therapies at least once per 91 days by the duration of preindex opioid prescribing, based on the number of patients receiving opioids. (a) No utilization. (b) Physical therapy. (c) Psychotherapy. (d) Psychosomatic care. (e) Special pain therapy (outpatient). (f) IMPT. Abbreviation: IMPT = interdisciplinary multimodal pain therapy.

**Figure:**
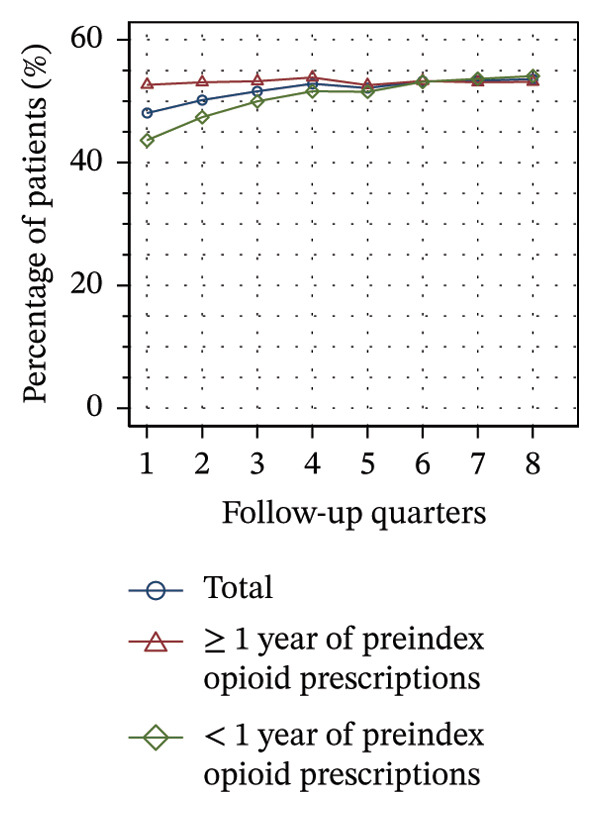
(a)

**Figure:**
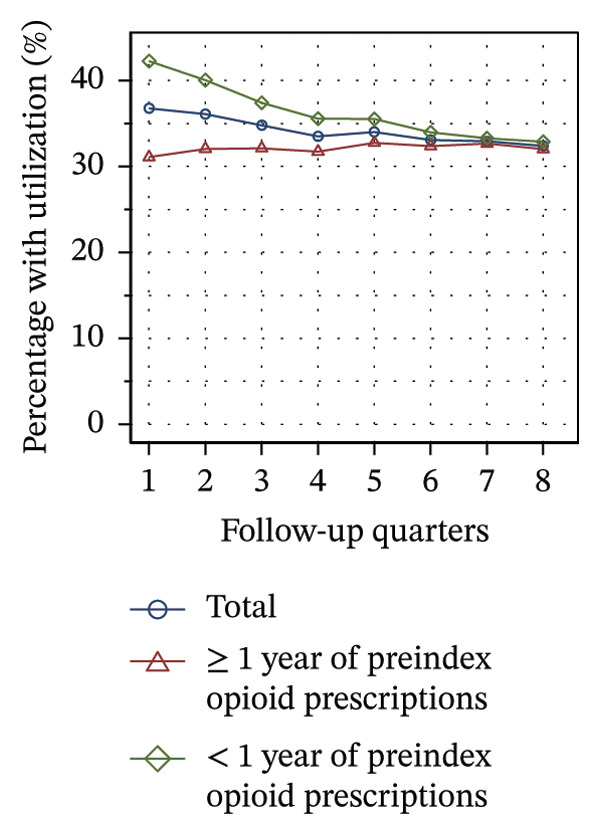
(b)

**Figure:**
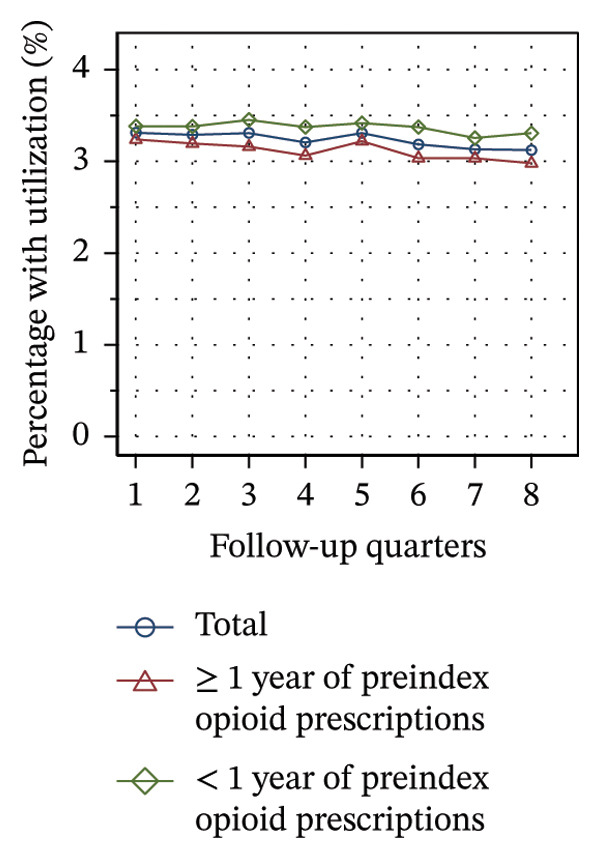
(c)

**Figure:**
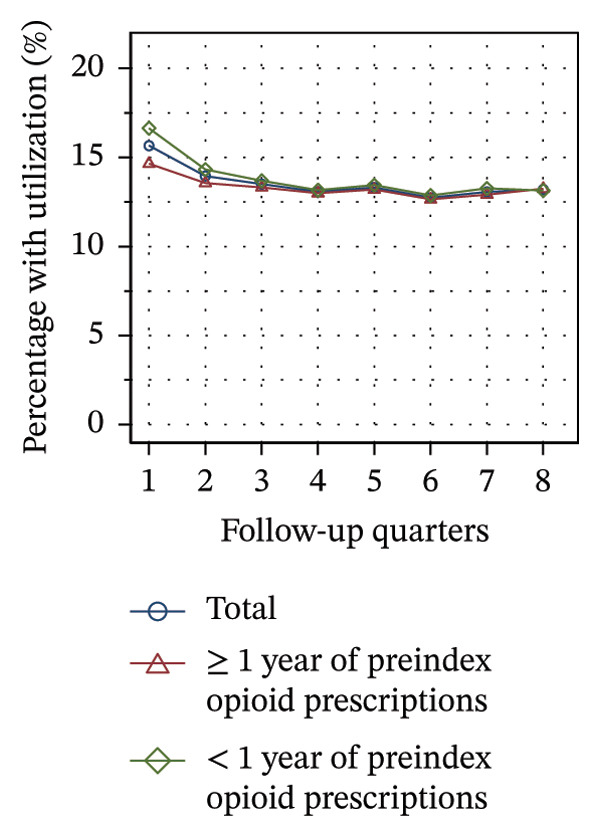
(d)

**Figure:**
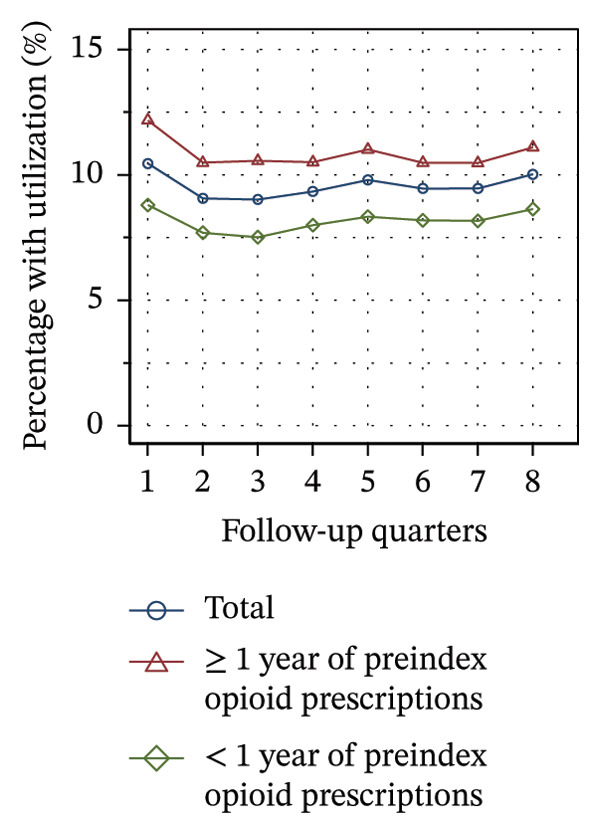
(e)

**Figure:**
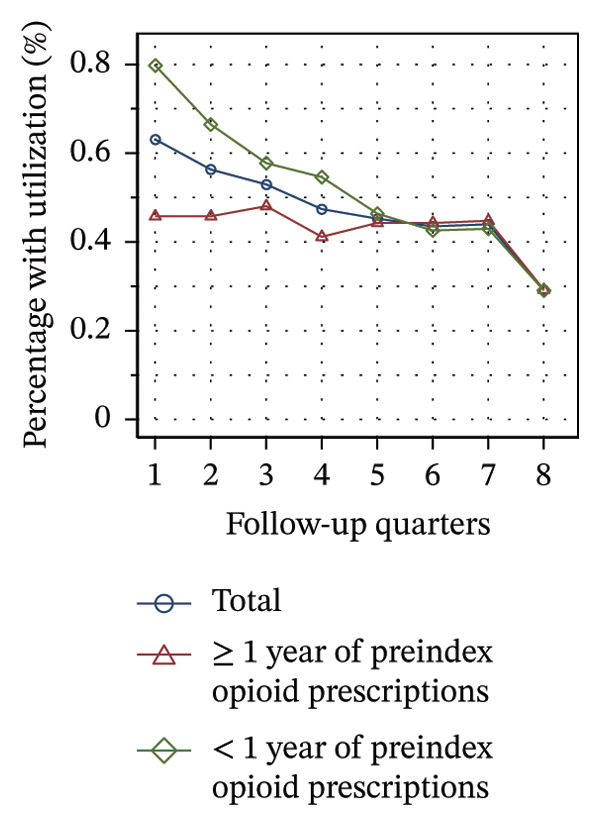
(f)

At the outset of the observation period, the proportion of patients receiving physical therapy was at 31% among patients with ≥ 1 year of preindex opioid prescriptions and remained on a consistent level throughout the observation period (see Figure 2(b)). The utilization rate for patients with < 1 year was substantially higher (42% in Q1) but exhibited a gradual decline until the final quarter, ultimately reaching the same level for both groups.

The provision of psychotherapy, psychosomatic care, and outpatient special pain therapy remained largely stable within both groups. Utilization of psychotherapy was slightly higher among patients with < 1 year of preindex opioid prescriptions throughout the observation period (e.g., 3.2% vs 3.4% in Q1, see Figure 2(c)), whereas utilization of psychosomatic care was higher in this group only during the first three quarters (15% vs 17% in Q1, see Figure 2(d)). With regard to special pain therapy, the utilization was higher among patients with ≥ 1 year of preindex prescriptions (12% vs 9% in Q1, see Figure 2(e)). The use of IMPT was higher among patients with < 1 year of preindex prescriptions during the first four quarters (e.g., 0.79% vs. 0.46% in Q1, see Figure 2(f)) and was similar in both groups during the last four quarters.

Figure 3 illustrates the service utilization within age groups over the course of the observation period. The highest utilization of physical therapy was observed in the two middle age groups (50–69 yrs: 31%; 70–89 yrs: 35% in Q8; see Figure 3(b)), whereas the youngest and oldest groups received the least (18–49 yrs: 26%; ≥ 90 yrs: 27% in Q8). The utilization of psychotherapy, psychosomatic care, special pain therapy, and IMPT exhibited a negative correlation with age, with the highest rates observed among the youngest age groups and the lowest rates observed among the oldest age groups. In particular, the utilization of psychotherapy (1.1% in Q1, see Figure 3(c)) and special pain therapy (1.8%, see Figure 3(e)) was negligible among the oldest patients. Individuals aged 18 to 49 used psychotherapies at notably higher frequency compared to the other age groups (10% in Q1, see Figure 3(c)). With the exception of physical therapy and IMPT, utilization of all treatments remained mostly steady within each age group.

FIGURE 3Utilization of nonpharmacologic therapies at least once per 91 days by age group, based on the number of patients receiving opioid prescriptions. (a) No utilization. (b) Physical therapy. (c) Psychotherapy. (d) Psychosomatic care. (e) Special pain therapy (outpatient). (f) IMPT. Abbreviation: IMPT = interdisciplinary multimodal pain therapy.

**Figure:**
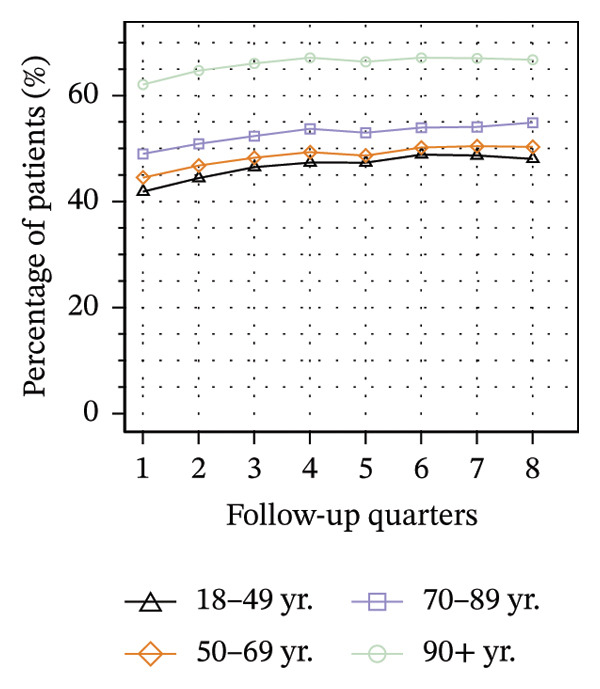
(a)

**Figure:**
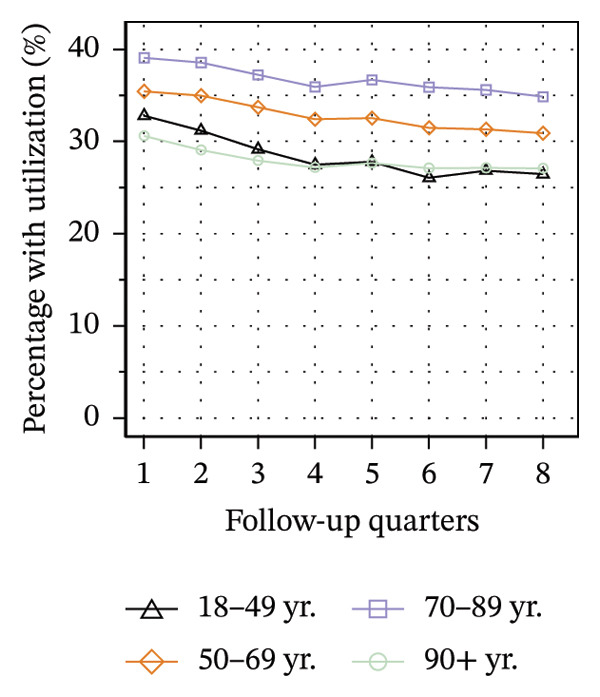
(b)

**Figure:**
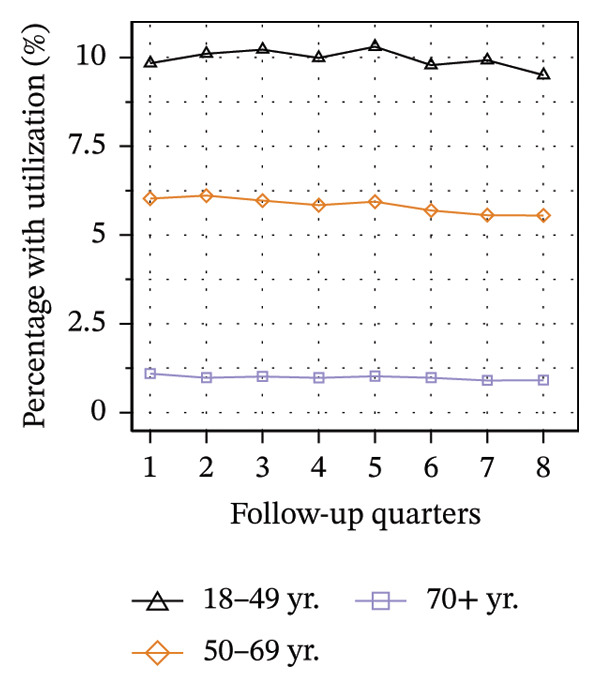
(c)

**Figure:**
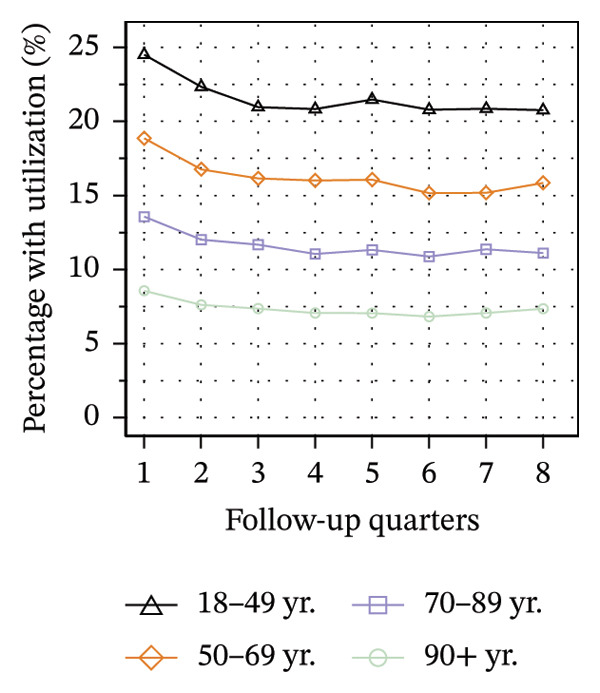
(d)

**Figure:**
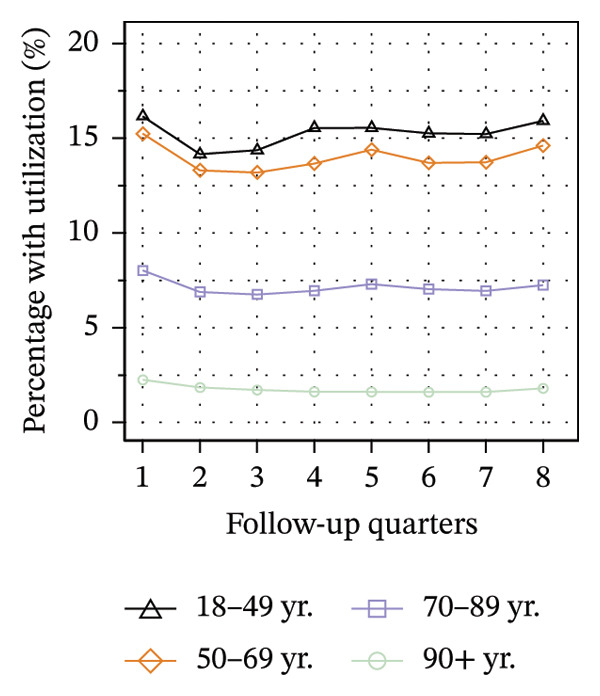
(e)

**Figure:**
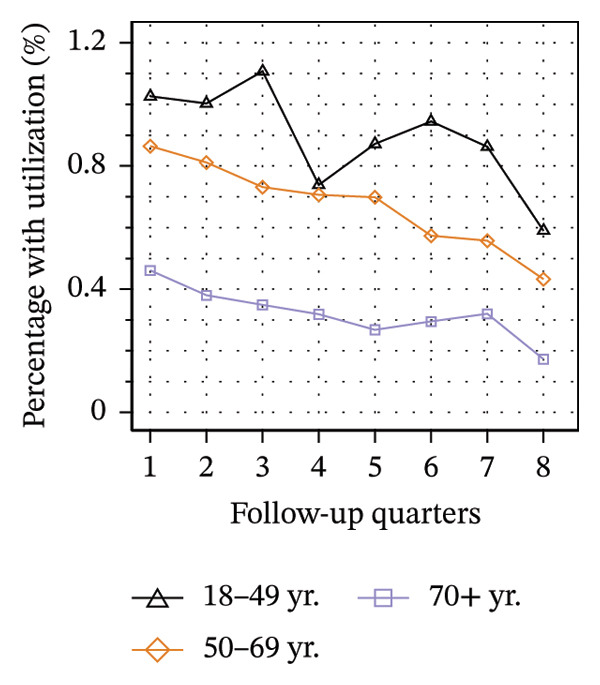
(f)

### 3.4. Service Utilization Within Diagnosis Groups

The provision of physical therapy was observed to be comparable across diagnoses, with the lowest rate observed for chronic pain disease (64%) and the highest rate for inflammatory rheumatic diseases (70%) (see Table 4). IMPT was most frequently used by patients with back pain, inflammatory rheumatic diseases, painful radiculopathy, and chronic pain disease (approx. 5%) and least frequently used by patients with osteoarthritis pain and (poly)neuropathy (approx. 3%). For the other treatments, a recurring pattern emerged among the diagnoses. The use of psychotherapy ranged from 7% among patients with osteoarthritis or polyneuropathy to 10% among those with painful radiculopathy or chronic pain disease. Psychosomatic care was utilized by 39% of patients with osteoarthritis pain or (poly)neuropathy, 45% of patients with chronic pain disease, and 47% of patients with painful radiculopathy. Outpatient special pain therapy was utilized between 18% (osteoarthritis pain) and 27% (painful radiculopathy) with regard to guideline‐based diagnoses, and 28% among patients with chronic pain disease. Notably, patients without any guideline‐based diagnosis or chronic pain disease in the first two quarters utilized clearly fewer services across all categories.

**TABLE 4:** Utilization over 2‐year observation period and mean age within diagnostic groups.

	**No diagnosis**	**Osteoarthritis pain**	**Back pain**	**Inflammatory rheumatic disease**	**(Poly)neuropathy**	**Painful radiculopathy**	**Chronic pain disease**
	**(*n* = 22,610)**	**(*n* = 46,084)**	**(*n* = 41,957)**	**(*n* = 38,058)**	**(*n* = 20,690)**	**(*n* = 19,200)**	**(*n* = 46,891)**
	**%**	**%**	**%**	**%**	**%**	**%**	**%**

Physical therapy	54.0	68.1	69.1	69.8	67.1	69.6	64.1
Psychotherapy	5.3	6.7	8.5	8.0	6.6	10.1	9.9
Psychosomatic care	26.9	39.1	43.6	42.8	38.5	47.2	44.9
Special pain therapy	6.0	18.2	24.0	23.1	19.8	27.4	28.2
IMPT	1.6	3.2	4.6	4.5	3.3	5.0	4.6

Mean age	70.9	75.0	71.8	73.0	73.3	70.0	70.6
SD	±16.3	±11.9	±13.1	±12.4	±12.4	±13.8	±14.2

Abbreviations: IMPT = interdisciplinary multimodal pain therapy, SD = standard deviation.

The regression models adjusted for comorbidity, age, gender, preindex opioid prescribing, and opioid therapy discontinuation indicated that associations between diagnostic groups and nonpharmacologic treatment utilization were statistically significant but weak in most cases (see Table 5; for full regression results, see Supporting Table 2). For instance, individuals diagnosed with chronic pain disease had 4% increased odds of provision of physical therapy (odds ratio (OR) = 1.04, *p* < 0.05), and those diagnosed with osteoarthritis pain had 10% increased odds of receiving psychotherapy (OR = 1.10, *p* < 0.001). Some diagnoses were more strongly associated with treatments, e.g., chronic back pain with a 50% increase in the odds of receiving special pain therapy (OR = 1.50, *p* < 0.001), and inflammatory rheumatic disease with a 53% increase in the odds of receiving IMPT (OR = 1.53, *p* < 0.001). Conversely, some negative associations were identified. These included the absence of one of the guideline‐based diagnoses, which reduced the odds of participation in psychosomatic care by 10% (OR = 0.90, *p* < 0.001), and a diagnosis of (poly)neuropathy, which was associated with a 7% reduction in the odds of receiving psychotherapy (OR = 0.93, *p* < 0.05). The association between a diagnosis of chronic pain disease and the provision of nonpharmacologic treatments was comparatively pronounced. The diagnosis of chronic pain disease increased the odds of receiving psychosomatic care by 51% (OR = 1.51, *p* < 0.001) and psychotherapy by 72% (OR = 1.72, *p* < 0.001), whereas the odds of receiving IMPT almost doubled (OR = 1.96, *p* < 0.001) and the odds of receiving special pain therapy more than quadrupled (OR = 4.20, *p* < 0.001).

**TABLE 5:** Logistic regression models examining associations between diagnostic groups and treatments over a 2‐year observation, adjusted for patient characteristics and comorbidity.

	**Physical therapy**	**Psychotherapy**	**Psychosomatic care**	**Special pain therapy**	**IMPT**
	**OR**	**OR**	**OR**	**OR**	**OR**

No guideline‐based diagnosis	1.00	0.94	0.90^∗∗∗^	1.11^∗∗^	1.00
Osteoarthritis pain	1.32^∗∗∗^	1.10^∗∗∗^	1.12^∗∗∗^	1.15^∗∗∗^	0.98
Back pain	1.19^∗∗∗^	1.10^∗∗^	1.17^∗∗∗^	1.50^∗∗∗^	1.40^∗∗∗^
Inflammatory rheumatic diseases	1.36^∗∗∗^	1.17^∗∗∗^	1.20^∗∗∗^	1.43^∗∗∗^	1.53^∗∗∗^
(Poly)neuropathy	1.21^∗∗∗^	0.93^∗^	0.98	1.28^∗∗∗^	0.99
Painful radiculopathy	1.21^∗∗∗^	1.18^∗∗∗^	1.25^∗∗∗^	1.39^∗∗∗^	1.23^∗∗∗^
Chronic pain disease	1.04^∗^	1.72^∗∗∗^	1.51^∗∗∗^	4.20^∗∗∗^	1.96^∗∗∗^

Elixhauser Comorbidity Index	1.07^∗∗∗^	1.08^∗∗∗^	1.10^∗∗∗^	0.99^∗∗^	1.08^∗∗∗^

McFadden’s pseudo‐*R* ^2^	0.04	0.12	0.06	0.15	0.07

*Note:* Control variables such as age, gender, preindex opioid prescribing, and discontinued opioid therapy; for full regression results, see Supporting Table 2.

Abbreviation: OR = odds ratio.

^∗^ *p* < 0.05.

^∗∗^ *p* < 0.01.

^∗∗∗^ *p* < 0.001.

Additional comorbidities were predominantly positively associated with the utilization of nonpharmacologic treatments. For example, the occurrence of one additional comorbidity increased the odds of utilizing physical therapy by 7% (OR = 1.07, *p* < 0.001), psychotherapy and IMPT by 8% (OR = 1.08, *p* < 0.001), and psychosomatic care by 10% (OR = 1.10, *p* < 0.001). In contrast, every additional comorbidity was associated with a 1% reduction in the odds of utilizing special pain therapy (OR = 0.99, *p* < 0.01). After adjustment for morbidity, age was significantly negatively associated with the utilization of treatments, except for physical therapy (see Supporting Table 2).

## 4. Discussion

### 4.1. Service Utilization

Of the 113,476 patients included in the study, approximately 23% did not receive selected nonpharmacologic treatments from SHI standard care over a two‐year period. Considering only those treatments explicitly recommended in the German guideline on LTOT for CNCP (physical therapy, psychotherapy, lifestyle modification), the proportion without utilization increases to about 36%. However, the guideline emphasizes that treatments recommended in other guidelines may be appropriate depending on the underlying pain condition [36]. In comparison, international findings vary: In Canadian studies defining chronic pain as pain persisting ≥ 3 months, 13%–14% reported not currently using either physical or psychological treatment for pain management [39, 40]. In an Australian cohort study of patients with CNCP and at least a 6‐week period of opioid use, 8% reported no utilization of physiotherapy, mental health, or specialist services at any time over a 4‐year period, whereas the annual estimates ranged from 25% to 36% [41].

In the same Australian cohort, physical therapy was used by 62% within 4 years, concurring with 62% within 2 years observed in our study. In contrast, our results indicate that 7% of patients received psychotherapy, whereas 27% in the Australian cohort reported the use of psychological treatment and 17% reported the use of psychiatric treatment within the 4‐year period. We also observed lower utilization rates for acupuncture (7%) compared to 21% identified by Hopkins et al. Special pain therapy was utilized by 16% and IMPT by 3% in our study; by comparison, 22% of the Australian cohort attended pain management programs [41]. However, a direct comparison of these studies should be made with caution, given differences in data collection methods, healthcare systems, service definitions, and a more elderly (median age 75 vs. 57 years) and more female population in our study (75% vs. 56%).

In principle, SHI in Germany offers comprehensive coverage, with modest copayments for therapeutic services and medications, and none for outpatient physician care. However, referrals to specialist care, such as outpatient pain therapy or IMPT, might be limited by their regional availability and accessibility [42]. For therapeutic services, such as physical therapy, shortages and waiting times have been identified as barriers to use in Germany [43]. International studies report the gatekeeper role of general practitioners [44] and a lack of confidence in nonpharmacologic treatments [45, 46] as limiting factors. The obstacles encountered by CNCP patients in the study by Hopkins et al. [44] led to frustration, with some patients even minimizing their pain management to the prescription of medication.

According to our findings, 23% of the study population received only opioids to manage pain, with no use of nonpharmacologic treatments. These findings may indicate a greater necessity for the expansion of spatial coverage [42], multidisciplinary resources [47, 48], and increased specialist training for physicians [48] to support the use of guideline‐recommended therapies. Further research is required to identify the patient‐ or supply‐side barriers that contribute to the nonuse of nonpharmacologic treatments by some opioid patients in Germany.

### 4.2. Utilization Over Time and by Patient Groups

The utilization of the majority of services remained largely constant throughout the observation period, with no service showing an upward trend. However, the total utilization within the 2‐year period was higher than in each of the follow‐up quarters, suggesting that many patients did not receive the treatments continuously or regularly. With the exception of outpatient special pain therapy, patients with < 1 year of preindex opioid prescriptions showed higher utilization across the service spectrum, indicating that patients were more likely to receive nonpharmacologic treatments at an earlier stage of opioid therapy. A US review from 2020 reports a reduction in future opioid prescriptions in patients with early nonpharmacologic treatment of musculoskeletal pain, but mainly refers to periods prior to the initiation of opioid therapy [49]. The use of nonpharmacologic interventions as first‐line therapy for CNCP is recommended in several evidence‐based opioid guidelines [15, 16, 50].

The use of physical therapy by patients with < 1 year of preindex opioid prescriptions decreased by nearly 10 percentage points over time. In contrast, Hopkins and colleagues did not find a significant correlation between time and the use of physical therapy among Australian cohorts, but did find a significant negative correlation for the use of specialized services [41]. There might be two overlapping interpretations of the decline observed in our study: First, the decline may be due to the discontinuation of opioid therapy in some of the patients who additionally received physical therapy, as this analysis was limited to patients with current opioid therapy during the time intervals. Second, physical therapy may have been discontinued, while opioid therapy was sustained. The first interpretation is consistent with the high proportion of patients with < 1 year of preindex prescriptions discontinuing opioid prescriptions over time (see Figure 1(a)), whereas the second is consistent with an increase in patients without utilization of nonpharmacologic treatments (see Figure 2(a)). Additionally, the increase indicates that at least some of the patients with continuing opioid therapy did not receive any further nonpharmacologic treatments besides physical therapy.

We identified a lower utilization of most services among older patient groups. A negative association between age and utilizing mental health services was also found among opioid patients in Australia [41]. In a cross‐sectional study conducted in Berlin among elderly individuals with CNCP and certified need of care, the use of physical therapy was found to be lowest in the oldest age group, which is consistent with our findings [51]. However, we also found that the youngest patients received less physical therapy than the middle‐aged groups. These trends are evident in the use of physical therapy in the general population of Germany, where the youngest and oldest age groups exhibit the lowest rates of utilization [52].

Depending on the diagnosis, the referral to physical therapy for SHI physicians is limited in Germany [53] and can be subject to efficiency evaluations [54]. A study by Kriegisch et al. suggested that adherence to the scheduled referral limit is associated with providers’ self‐reported dissatisfaction with their pain management [48]. The 2021 reform of German prescription regulations facilitated regular referrals for physical therapy [55], and therefore, potential changes in treatment utilization could not be accounted for in our data. Nevertheless, our results may indicate a potential need for further adjustments in the regulatory framework to provide continuous physical therapy to chronic pain patients. Moreover, an inconsistent utilization over time may emphasize the necessity for discharge strategies that improve patients’ self‐management of pain [56]. The lower utilization by elderly individuals raises the question of potential barriers to the use of nonpharmacologic therapies among certain patient groups.

### 4.3. Utilization by Diagnostic Group

The use of nonpharmacologic therapies was mostly comparable between the diagnosis groups. However, the two diagnosis groups with the highest mean age (osteoarthritis, polyneuropathy) tended to have lower utilization and the diagnosis groups with the lowest mean age (painful radiculopathy, chronic pain disease) tended to have higher utilization. This is noteworthy as it corroborates our previously reported results indicating lower utilization among older patients. Consistent with our descriptive results, we found that after adjusting for age, gender, comorbidity, and other variables, the diagnostic groups were predominantly weak predictors for the utilization of nonpharmacologic therapies. And vice versa, adjusting for morbidity, older age was still associated with a lack of utilization.

It is noteworthy that individuals without a guideline‐based diagnosis were consistently the least frequently treated with nonpharmacologic interventions. However, after adjustment for covariates, there was a significant association with the use of outpatient special pain therapy. This might indicate a heterogeneous composition of pain indications not included in the guideline but associated with the treatment in a specialized pain program.

The chronic pain disease was frequently diagnosed and comparably strongly associated with the use of nonpharmacologic treatments, especially multimodal programs. This could suggest both a high need for treatment among patients with chronic pain disease and a frequent usage of the diagnosis within specialized services.

The magnitude of the observed effects may be limited by the use of nondisjoint diagnostic groups and thus heterogeneous reference categories. We conclude that unobserved factors and, except for physical therapy, younger age may be stronger predictors for the use of most nonpharmacologic therapies than specific diagnoses. This indicates barriers to treatment for elderly patients. Future research should characterize diagnostic groups among LTOT patients in more detail, including diagnosis‐specific utilization of nonpharmacologic treatments, and examine unobserved factors. For example, in Hopkins et al. [41], pain interference was associated with specialized and mental health service use.

### 4.4. Limitations

Our study based on SHI claims data has some limitations. These are as follows:

• We only included patients from one SHI provider, which may limit the external validity. Nevertheless, since the *DAK-Gesundheit* is a large, nationwide SHI provider, a substantial number of subjects could be enrolled and selection bias could be minimized. In addition, all German SHI funds provide the same range of services as part of the SHI standard care.
• Administrative SHI claims data capture services as charged. Consequently, clinical parameters (e.g., indication, adherence, response) and what was actually performed in individual cases (e.g., psychosomatic care, special pain therapy, multimodal therapy) cannot be directly observed. Billed services are used as a proxy for service utilization.
• Our selection of relevant nonpharmacologic treatments may not be exhaustive. Notably, our data set lacked representation of lifestyle modifications and self‐help services. Improvements in pain and function have also been shown in some cases for self‐directed physical activities and exercises [57]. Nevertheless, our study covers a comprehensive range of complex and multimodal therapies.
• Not all of the nonpharmacologic treatments and services may primarily target CNCP (e.g., psychotherapy). ICD‐10 diagnoses used for grouping may not directly correspond to the indication for the index opioid prescription.
• Utilization was examined on the basis of broad time periods (once within 2 years, once within 90 days), which may not reflect continuity of service provision.

## 5. Conclusions

Our findings indicate that a notable proportion of patients with CNCP receiving LTOT had no documented nonpharmacologic treatments, despite guideline recommendations that opioid therapy should be complemented by additional modalities. Nonpharmacologic treatments were utilized more frequently by patients who had been on opioid therapy for a shorter period of time, with the use decreasing over time for some treatments. It is noteworthy that among elderly patients, the majority of treatments were used less frequently, even after adjusting for morbidity. Recognizing that treatment selection typically depends on the diagnosis, the largest diagnostic group with the most nonspecific diagnosis—chronic pain disease—showed the highest proportions of specialized pain therapy. Other diagnostic groups were only weakly associated with treatment utilization.

The results suggest that the utilization of nonpharmacologic interventions may be limited at older ages and after longer periods of treatment. Currently, unobserved barriers to the use of nonpharmacologic treatments require further investigation.

## Funding

This study was entirely funded by the Innovation Fund of the German Federal Joint Committee (G‐BA; funding No. 01VSF19059).

Open Access funding enabled and organized by Projekt DEAL.

## Disclosure

The funding body has in no way influenced the design of the study, the collection, analysis, and interpretation of data or the writing process of this manuscript.

## Conflicts of Interest

Cordula Riederer is employed by the statutory health insurance company *DAK-Gesundheit*. Joachim Nadstawek (chairman) and Wolfgang Straßmeir are members of the Association of German Doctors and Psychotherapists practicing in Pain Medicine and Palliative Care (BVSD e.V.). The other authors declare no conflicts of interest.

## Supporting Information

Supporting 1. Supporting Figure 1: Selection, observation period, and preindex prescriptions.

Supporting 2. Supporting Table 1: Evidence‐ and consensus‐based diagnoses for potential opioid use according to the German LTOT guideline.

Supporting 3. Supporting Table 2: Full results of logistic regression models examining associations between diagnostic groups and treatments over the 2‐year observation period.

## Supporting information


**Supporting Information** Additional supporting information can be found online in the Supporting Information section.

## Data Availability

In accordance with Sections 67b and 75 of Book X of the German Code of Social Law, the German Federal Office for Social Security (Bundesamt für Soziale Sicherung, BAS) has approved the transfer of social security data in November 2021 (§67b and §75 SGB X). Due to data privacy regulations, the data cannot be made available.
