# Supplementary material for: A Large Retrospective Observational Study of Nonpharmacologic Treatment Use Among German Patients Receiving Long‐Term Opioid Therapy for Chronic Noncancer Pain
Source: Pain Res Manag. 2026 Apr 29;2026:2607538. doi: 10.1155/prm/2607538 (PMC13126428; doi:10.1155/prm/2607538)
Supplement: Supplementary file 1 — Supporting Information Additional supporting information can be found online in the Supporting Information section. [file PRM-2026-2607538-s001.pdf]

Supplementary material

Supplementary Figure 1: Selection, observation period, and pre-index prescriptions

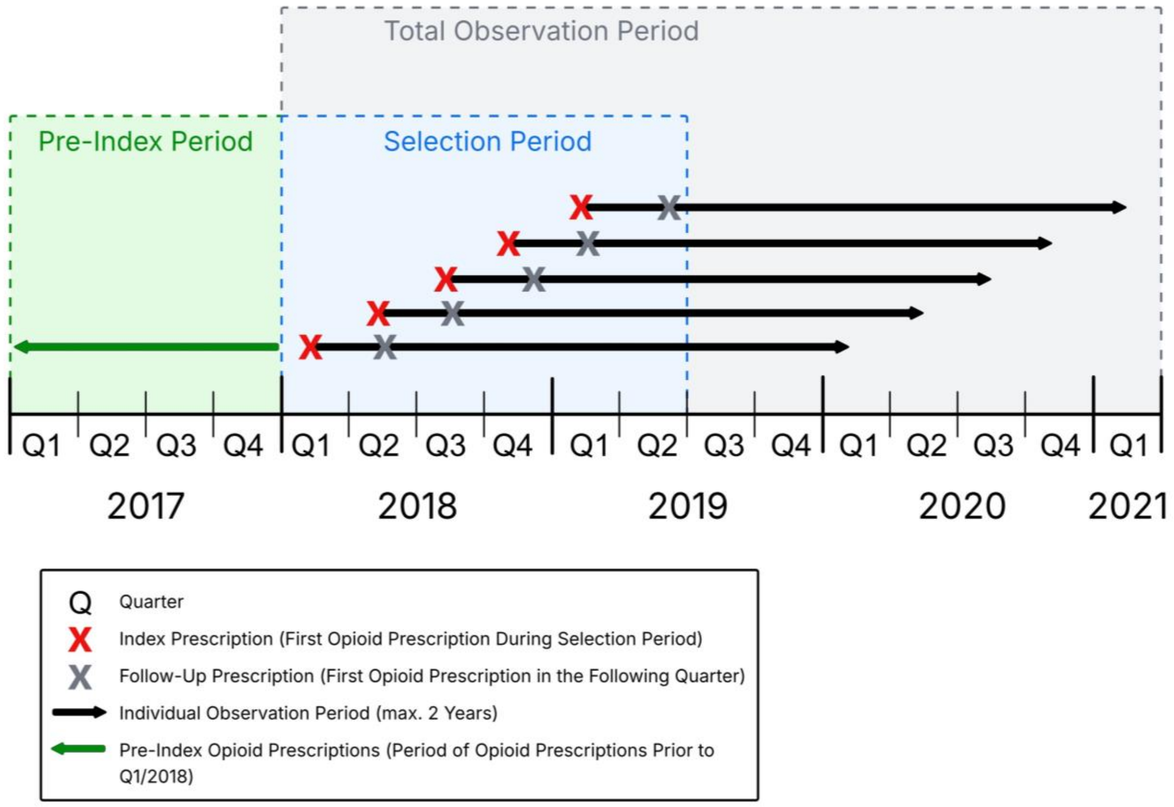

Pain Research and Management – A large retrospective observational study of nonpharmacologic treatment use among German patients receiving long-term opioid therapy for chronic non-cancer pain

Supplementary Table 1: Evidence- and consensus-based diagnoses for potential opioid use according to German LTOT guideline

No.	Indication	Diagnosis - ICD-10-GM
**1**	Chronic osteoarthritis pain	M15-M19
**2**	Chronic back pain	M42.16-M42.19, M42.90, M42.96-99, M43.0, M43.1, M47.26, M47.27, M47.29, M47.86, M47.87, M47.88, M47.99, M48.06, M48.2, M54.16, M54.5, M55.3, M.99.33; M99.43, M99.53
**3**	Chronic pain associated with inflammatory rheumatic diseases other than rheumatoid arthritis (e.g., systemic lupus erythematosus, spondyloarthritis)	M32.- M45-M49
**4**	**(Poly)neuropathy**	
	Diabetic polyneuropathy	G63.2 in combination with E10-E14
	Polyneuropathy of etiology other than diabetes	G60-G64 (without G63.2)
	Traumatic trigeminal neuropathy	G50.9
	Central (cerebral) neuropathic pain (e.g., after thalamic infarction, multiple sclerosis)	D33.-, D43.-, G04.8, G04.9, G05.-, G06.-, G07, G35.-, G37.3, G37.4, I60.-, I62.-, I63.-, Q27.3, Q28.-, S06.-,
	Secondary headache and central (cerebral) neuropathic pain (e.g., after thalamic infarction, multiple sclerosis)	G44.8 in combination with (D33.-, D43.-, G04.8, G04.9, G05.-, G06.-, G07, G35.-, G37.3, G37.4, I60.-, I62.-, I63.-, Q27.3, Q28.-, S06.-)
**5**	Painful radiculopathy	M54.1
**6**	Rheumatoid arthritis with persistent pain	M06.-
**7**	Postzoster neuralgia	B02.2, G53.0
**8**	Phantom Pain	G54.6
**9**	Pain after spinal cord injury	S14.0, S14.1, S14.7, S19.80, S24.0, S24.1, S24.2, S24.7, S29.8, S34.0, S34.1, S34.2, S34.3, S34.7, S39.81
**10**	Restless legs syndrome	G25.81
**11**	Parkinson syndrome	G20, G21
**12**	Fibromyalgia Syndrome	M79.70
**13**	Chronic pain in manifest osteoporosis (vertebral body fractures)	M80.08, M80.18, M80.28, M80.38, M80.48, M80.58, M80.88, M80.98
**14**	Chronic extremity pain in ischemic and inflammatory arterial occlusive disease	I70, I73, I77, I79
**15**	Chronic pain in decubitus grade 3 and 4	L89.2-, L89.3-
**16**	Chronic fixed contracture pain in patients in need of long-term care	M24.5, M67.1
**17**	Chronic complex regional pain syndrome type I and II	G56.4/G57.8/ M89.0 (until 2018), G90.5-G90.7 (since 2019),
**18**	Chronic lower abdominal pain in women with marked adhesions and/or multilocular endometriosis	N73.6, N80.-

Adopted from German LTOT guideline (see references [15, 35])

Supplementary Table 2: Full results of logistic regression models examining associations between diagnostic groups and treatments over the two-year observation period

	**Physical therapy**	**Psycho-therapy**	**Psycho-somatic care**	**Special pain therapy**	**IMPT**
	**OR**	**OR**	**OR**	**OR**	**OR**
Intercept	1.10**	1.95***	2.73***	0.64***	0.13***
Age (in years)	1.00***	0.94***	0.97***	0.96***	0.96***
Female	1.53***	1.52***	1.53***	1.10***	1.34***
Long-term prescription history	0.66***	0.86***	0.87***	0.93***	0.80***
Discontinued opioid therapy	1.38***	1.11***	1.11***	0.94**	1.19***
ICD-10 diagnoses					
No guideline-based diagnosis	1.00	0.94	0.90***	1.11**	1.00
Osteoarthritis pain	1.32***	1.10***	1.12***	1.15***	0.98
Back pain	1.19***	1.10**	1.17***	1.50***	1.40***
Inflammatory rheumatic diseases	1.36***	1.17***	1.20***	1.43***	1.53***
(Poly)neuropathy	1.21***	0.93*	0.98	1.28***	0.99
Painful radiculopathy	1.21***	1.18***	1.25***	1.39***	1.23***
Chronic pain disease	1.04*	1.72***	1.51***	4.20***	1.96***
Elixhauser Comorbidity Index	1.07***	1.08***	1.10***	0.99**	1.08***
McFadden's Pseudo R^2	0.04	0.12	0.06	0.15	0.07

OR Odds ratio

*** $p < .001$; ** $p < .01$; * $p < .05$;
